# Cervical subtotal discectomy prosthesis validated in non-human primate model: A novel artificial cervical disc replacement concept?

**DOI:** 10.3389/fbioe.2022.997877

**Published:** 2022-10-13

**Authors:** Yang Liu, Jin Wo, Haoran Zhu, Zhonghai Huang, Pan Zhou, Jinpei Yang, Shuai Zheng, Libing Zhou, Fengjin Tan, Guodong Sun, Zhizhong Li

**Affiliations:** ^1^ Department of Orthopedics, First Affiliated Hospital and Fifth Affiliated Hospital, Jinan University, Guangzhou, China; ^2^ Guangdong-Hongkong-Macau Institute of CNS Regeneration, Ministry of Education CNS Regeneration Collaborative Joint Laboratory, Jinan University, Guangzhou, China; ^3^ Huizhou Third People’s Hospital, Guangzhou Medical University, Huizhou, China; ^4^ Orthopedics and Traumatology, Yantai Hospital of Traditional Chinese Medicine, Yantai, China

**Keywords:** cervical artificial disc replacement, cervical arthroplasty, artificial disc, prosthesis, primate model, biomechanics

## Abstract

**Objective:** To evaluate the biological function of cervical subtotal discectomy prosthesis (CSDP) implantation in a non-human primate model.

**Methods:** A CSDP was tested for cytocompatibility and osseointegration capacity before implantation in non-human primates. Subsequently, the CSDP was improved based on three-dimensional CT measurements of the non-human primate cervical spine. Eight cynomolgus monkeys were selected for removal of the intervertebral disc and lower endplate of the C5/6 segment to complete the model construction for CSDP implantation. In 18-month follow-up, physiological indices, radiology, and kinematics were assessed to estimate the biological function of the CSDP in non-human primates, including biosafety, osseointegration, and biomechanics.

**Results:** Co-cultured with the CSDP constituent titanium alloy (Ti6Al4V-AO), the mouse embryo osteoblast precursor cell MC3T3-E1 obtained extended adhesion, remarkable viability status, and cell proliferation. After implantation in the mouse femur for 28 days, the surface of Ti6Al4V-AO was covered by a large amount of new cancellous bone, which formed further connections with the femur cortical bone, and no toxicity was detected by blood physiology indices or histopathology. After completing implantation in primate models, no infection or osteolysis was observed, nor was any subsidence or displacement of the CSDP observed in CT scans in the 18-month follow-up. In particular, the interior of the cervical vertebra fixation structure was gradually filled with new trabecular bone, and the CSDP had achieved fixation and bony fusion in the vertebral body at 1 year post-operation. Meanwhile, no signs of inflammation, spinal cord compression, adjacent segment degeneration, or force line changes were observed in subsequent MRI observations. Moreover, there were no pathological changes of the joint trajectory, joint motion range, stride length, or the stance phase ratio revealed in the kinematics analysis at 3, 6, 12, or 18 months after CSDP implantation.

**Conclusion:** We successfully designed a new cervical subtotal discectomy prosthesis and constructed an excellent non-human primate implantation model for the evaluation of subtotal disc replacement arthroplasty. Furthermore, we demonstrated that CSDP had outstanding safety, osseointegration capacity, and biomechanical stability in a non-human primate model, which might be a new choice in the treatment of cervical disc diseases and potentially change future outcomes of degenerative cervical diseases.

## 1 Introduction

The emergence of artificial cervical discs has advanced the surgical treatment of the cervical intervertebral disc, that is, cervical artificial disc replacement (CADR) treatment for degenerative cervical diseases. The gold standard for CADR, which preserves the motion of the operated segment and the compensating mobility of the adjacent segment, had positive clinical and functional outcomes compared with anterior cervical discectomy and fusion (ACDF). Interestingly, most of the artificial discs currently approved by the Food and Drug Administration (FDA) are total disc-replacement prostheses that require the removal of both the upper and lower endplates during implantation. Nevertheless, excessive surgical resection and prosthesis design flaws may lead to uneven stress distribution and localized stress concentration at the bone interface, thereby increasing the risk of prosthesis failure (Anderson and Rouleau, 2004; Thaler et al., 2013). Although attempts have been made to prevent such failure in artificial cervical discs via surface modification at the interface of the upper and lower endplates, the practical results appear unsatisfactory. Additionally, the occurrence of chronic problems, such as hypermobility and heterotopic ossification of the operated segment, has been noted (Di Martino et al., 2015).

To this end, we designed a new prosthesis named cervical subtotal discectomy prosthesis (CSDP) based on the concept of “hemiarthroplasty” to reduce CADR complications while preserving the upper endplate at the time of implantation. It consists of a cervical disc prosthesis (CDP) structure, cervical vertebra fixation (CVF) structure, link structure, and locking screw. The specially designed ellipsoid-in-socket articulation, composed of a CDP structure and a link structure, endows the CSDP with a self-limiting motion function. In addition to providing the ellipsoidal articulation of the CSDP, the other end of the link structure is firmly anchored to the CVF structure by the locking screw. It is noteworthy that the fusion of the CVF structure with the vertebra is the foundation of the long-term stability of the CSDP.

Like hemiarthroplasty, CSDP implantation requires the removal of fewer cervical structures to complete disc replacement. It is well known that reducing the removal of one endplate reduces the risk of prosthesis subsidence; however, it may weaken the overall stability of the cervical spine sequence. In our previous research (Wo et al., 2021), we preliminarily proved by finite element analysis that the CSDP had excellent mechanical stability. However, there are limitations to the computer simulation verification. In contrast to the default in finite element analysis, in which the vertebra and prosthesis are tightly integrated, the bone-implant interface *in vivo* is much more complicated, due, for instance, to the existence of bone-implant interface micromotion (Rahimizadeh et al., 2018). Whether osseointegration of the bone-implant interface occurs determines the overall stability of the CSDP, and the CVF structure is critical for the mechanical settlement of CSDP. Therefore, as a newly designed artificial disc, it is essential to evaluate the CVF structure and overall performance of the CSDP in animals.

In this study, we constructed a non-human primate model of CSDP implantation and performed a comprehensive evaluation of the safety and biomechanics of the prosthesis. Before implantation, we verified the cytotoxicity and osteointegration of CSDP materials primarily at the cellular level and in a mouse model. Based on the three-dimensional CT measurement of the cervical spine of cynomolgus monkeys, the CSDP was improved and then schematically implanted in C5/6 of the non-human primate model. Additionally, physiological indices, radiology, and kinematics analyses were assessed in the 18-month follow-up.

## 2 Materials and methods

### 2.1 Materials and reagents

Mouse embryo osteoblast precursor cells (MC3T3-E1) were purchased from Shanghai Zhongqiao Xinzhou Biotechnology Co., Ltd. (Shanghai, China). The CSDP, metal pins (specification, 0.5 × 5 mm) and discs (diameter, 8 mm) of titanium alloy (Ti6Al4V-AO, Ti6Al4V), were produced by Jiangsu Brightness Medical Devices Co., Ltd. (Jiangsu, China). The Ti6Al4V-AO, which was coated with an oxide modification layer for CSDP construction, was modified from a titanium alloy, Ti6Al4V, by surface anodic oxidation. The standard culture medium was minimum Eagle’s medium alpha (MEM-α, Thermo Fisher, United States) containing 10% fetal bovine serum (Euroclone), 100 U/mL penicillin, and 100 mg/ml streptomycin. The Cell Counting Kit-8 (CCK-8), purchased from DOJINDO, Japan, was used for estimating the viability and proliferation of the MC3T3-E1 cells co-cultured with Ti6Al4V-AO and Ti6Al4V metal discs. The LIVE/DEAD reagent (L7010, Invitrogen), purchased from Thermo Fisher Scientific Inc., was used to quantify the viability of MC3T3-E1 cells co-cultured with Ti6Al4V-AO and Ti6Al4V metal pins.

### 2.2 Animals

C57BL/6J mice (female, 20–25 g) were purchased from Guangdong Medical Experimental Animal Center (Guangzhou, China). All the mice were maintained and propagated under pathogen-free conditions at the Animal Management Center of the Jinan University Laboratory, at a temperature of 22–25°C, relative humidity of 55% ± 5%, and a 12 h light/12 h dark cycle, with adequate feed and sterilized drinking water. Cynomolgus monkeys (male, 9–12 years, and 9.45–10.20 kg) were purchased from Huazhen Biotechnology Co., Ltd. All monkeys were fed in a single cage at a temperature of 2l–25°C, relative humidity of 40%–60%, ventilation of 8 times/h, and a 10 h light/14 h dark cycle, and interactive activities with neighboring monkeys were maintained pre- and postoperatively. Experimental ethics were approved by the Committee on the Management and Use of Laboratory Animals.

### 2.3 Cervical subtotal discectomy prosthesis design

**FIGURE 1:**
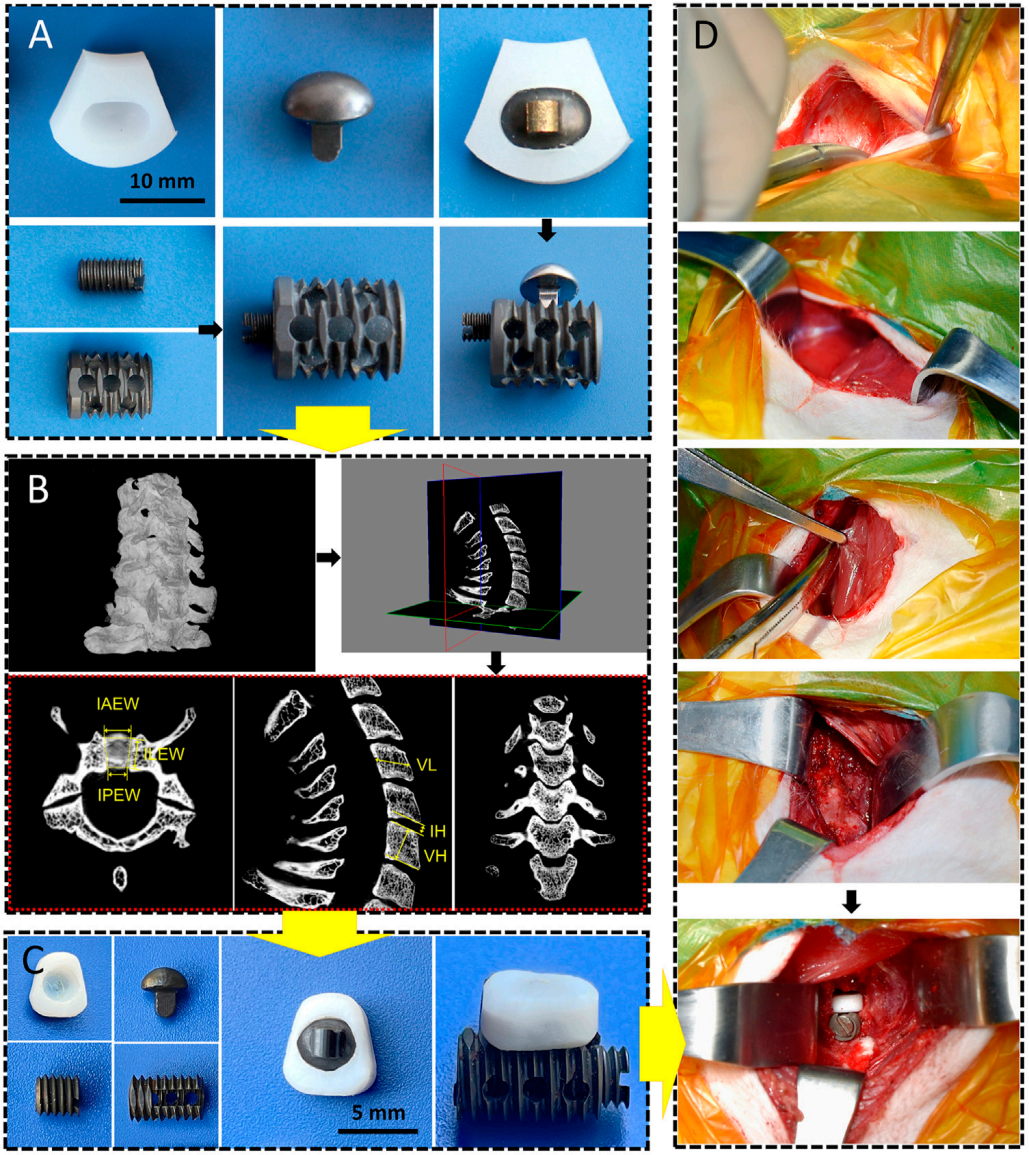
CSDP design, improvement, and implantation. **(A)** The cervical subtotal discectomy prosthesis (CSDP) design. Four components: CDP structure, link structure, CVF structure, and locking screw. **(B)** Cervical spine micro-CT anatomical measurements in transverse, sagittal, and coronal planes. IAEW, intervertebral anterior edge width. IPEW, intervertebral posterior edge width. ILEW, intervertebral lateral edge width. IH, intervertebral height. VH, vertebral height. VL, vertebral length. **(C)** Improved CSDP for non-human primate. **(D)** Construction of non-human primate model of CSDP implantation.

The CSDP is composed of four components: a CDP structure (ultrahigh-molecular-weight polyethylene [UHMWPE]), link structure, CVF structure, and locking screw (titanium alloy). As an artificial cervical disc, the key to CSDP with motor function is the construction of ellipsoid-in-socket articulation. This ellipsoidal artificial joint, consisting of the ellipsoidal convex handle of the link structure with the arc dome semi-surrounded by the CDP structure, was designed to balance mobility in all directions with limited range of motion (ROM) in flexion and extension (15°), lateral flexion (5°), and rotation (4°). In contrast to previous artificial discs with ball-in-socket articulation designs, the ellipsoidal joint allows the CSDP to be restricted from hypermobility during rotation, which manifests as self-locking. In addition, the link structure was designed to affix the CDP structure tightly to the CVF structure using the locking screw. The long-term biochemical stability of the CSDP depends on the fixation of the CVF structure in the vertebra. Thus, the CVF structure was anodized to increase its osteogenic capability and designed as a hollow cylinder with a threaded surface to promote the ingrowth of new cancellous bone (Figure 1A). Before CSDP implantation, a groove penetrating the upper endplate of the surgical vertebra was made using a high-speed burr and curettage. Then, the CVF structure was screwed into the groove, followed by locking of the CDP structure for early fixation.

### 2.4 Biocompatibility evaluation of cervical subtotal discectomy prosthesis *in vitro*

The UHMWPE, which constitutes the CSDP, has been extensively recognized for its good biocompatibility (Macuvele et al., 2017). Therefore, the safety evaluation of Ti6Al4V-AO metal, another constituent material, is of paramount importance. To better understand the biocompatibility of the CSDP, Ti6Al4V-AO metal was assessed for cytocompatibility in MC3T3-E1 cells and for cytotoxicity in the LIVE/DEAD assay.

#### 2.4.1 Cell culture and inoculation

MC3T3-E1 cells were cultured in cell dishes at 37°C, 5% CO_2_, with MEM-α being replaced every 3 days. Before cell seeding, all sterilized Ti6Al4V-AO and Ti6Al4V metal discs were transferred into 24-well plates for pre-moistening with MEM-α for 24 h. Then, MC3T3-E1 cells were inoculated on the samples (1×10^4^ cells/disc) and cultured for 5 h. The disc samples were washed twice with phosphate buffer solution (PBS; Thermo Company, United States) and transferred into new 24-well plates with MEM-α solution for long-term culture.

#### 2.4.2 Cell morphology observation

After incubation for 1, 3, and 7 days, the metal samples were taken out and washed twice with PBS and then placed in 2.5% glutaraldehyde for cell immobilization at 4°C for 24 h. After aspirating the fixative and rinsing the samples with PBS (15 min, 3 times), 30%–95% gradient alcohol was used to dehydrate the samples for 15 min each, and absolute ethanol twice for 20 min. Finally, after critical point drying and spraying of the gold coating, cell–metal samples were prepared for scanning electron microscopy (SEM).

#### 2.4.3 Cell viability estimation

After incubation for 1, 3, and 7 days, the metal samples were transferred to a new 24-well plate, then 2 ml of CCK-8 (10% concentration) was added to each well and cultured at 37°C for 1 h. Subsequently, 100 μL solution per well was extracted into a new 96-well plate, then the absorbance (optical density, OD) was measured at 450 nm using a microplate reader, and the cell viability was calculated additionally.

#### 2.4.4 LIVE/DEAD assay of cytotoxicity

The MC3T3-E1 cells were cultured overnight in 12-well plates at a density of 5.0×10^4^ cells/well, followed by co-cultivation with metal pins (Ti6Al4V-AO or Ti6Al4V) for 1, 3, and 7 days at 37°C. For the LIVE/DEAD assays, the co-cultured cells were stained using a LIVE/DEAD (green/red) reagent, placed in the dark for 30 min, and then examined using an inverted fluorescence microscope (Axio Observer A1; Carl Zeiss).

### 2.5 Osseointegration evaluation of cervical subtotal discectomy prosthesis

To better understand the biocompatibility and osseointegration of CSDP *in vivo*, metal pins made of the same material as the CSDP were implanted in mouse femurs, with histopathology, blood physiological indices, and osseointegration capacity determined at l4 and 28 days postoperatively.

#### 2.5.1 Establishment of the intramedullary nail implantation model

A total of 24 C57BL/6J female mice were randomized into the Ti6Al4V-AO group and the Ti6Al4V control group. After intraperitoneal anesthesia (l.25% tribromoethanol, 0.2 ml/10 g), the left knee was shaved and disinfected with iodine, and a 5-mm incision was made in the middle of the knee, exposing the patellar ligament. A 26G syringe needle was inserted in the left femur, through the lateral midpoint of the patellar ligament parallel to the femoral shaft, and the medullary cavity was reamed. The metal pin was then implanted in the femur after being reamed with 23G and 21G syringe needles; this was followed by incision washing, iodophor disinfection, and incision suturing.

#### 2.5.2 Determination of physiological indices

Whole-blood samples from mice were collected at l4 and 28 days post-operation, under fasting conditions for 12 h before blood collection. After puncturing through the left ventricle, blood (0.5 ml) was extracted and preserved in a collection tube, to which EDTA was added for anticoagulation, and was analyzed with a KX-21N automatic blood analyzer. The blood physiological indices included white blood cells (WBCs), red blood cells (RBCs), and platelets (PLTs). Blood levels of alanine aminotransferase (ALT) and aspartate aminotransferase (AST) were used as liver function markers, while blood urea nitrogen (BUN) was used as a kidney function marker. After the mice were harvested, their major organs, such as the heart, lung, liver, kidney, and spleen, were immediately removed to prepare frozen sections, and the left femurs were removed and placed in paraformaldehyde fixative for micro-CT examination. Subsequently, the metal implants were removed to prepare the bone tissue sections. Both organ and bone tissue sections were stained with hematoxylin and eosin, sliced at a thickness of 6 μm, and then observed under an optical microscope.

#### 2.5.3 Observation of bone morphology

To observe the morphology of the new bone around the implant, micro-CT (SkyScan1276, Bruker Corporation, United States) was used to perform sequential thin-layer scanning (12 μm) of the femur mentioned above. The scanning parameters were set as follows: source voltage, 100 kV; source current, 200 μA; reference intensity, 58000; exposure, 408 mS; rotation step, 0.40°; image pixel size, 12 μm; and scaled image pixel size, 12 μm. To minimize metal artifacts near the metal pin, a filter of Al and Cu was used. In addition, the reconstruction thresholds for CS-to-image conversion were set to 0.0 for the minimum and 0.08 for the maximum. The original CT data were then imported into Mimics (version 20.0), with the mask of the implant established by adjusting the threshold range (35–150, 0–255 in total), and the three-dimensional (3D) model of the metal pin (white color) was calculated and reconstructed. Similarly, the mask (threshold range: 240–255, 0–255 in total) and the 3D model (red color) of the new bone around the implant were established and calculated, with the obvious connection to the cortical bone removed. Finally, the morphological differences of the new bone were compared by displaying the 3D images of the metal implants and new bone simultaneously.

### 2.6 Cervical subtotal discectomy prosthesis improvement

The size of the CSDP applied to non-human primates influences the success of surgery and long-term postoperative stability. The most appropriate CSDP size is anatomically adapted, which poses a challenge for the previous construction of CSDPs of human size. To this end, we performed anatomical measurements of the cervical spine of cynomolgus monkeys to precisely improve the CSDP to fit the individuals’ physiological sizes.

#### 2.6.1 Cervical spine measurement in non-human primates

Cervical spine specimens were all taken from cynomolgus monkeys (male, weighing 8–11 kg) that died naturally. A total of 25 monkeys were selected for whole-cervical spine sampling, and the muscles and soft tissues around the vertebrae were peeled off. Before micro-CT measurement, all specimens were excluded for bone diseases, such as trauma, deformity, and tumor, using radiography. The cervical spines of specimens 2–7 were scanned by micro-CT in a layer of l8 µm, and the data were imported into Mimics (version 20.0). Then, 3D models of the C2–C7 cervical vertebrae were reconstructed after creating and calculating the mask of the vertebra by adjusting the bone threshold range. The CT data were imported into SkyScan software, and the intervertebral anterior edge width (IAEW), intervertebral posterior edge width (IPEW), and intervertebral lateral edge width (ILEW) were subsequently measured in the axial view; the intervertebral height (IH), vertebral height (VH), and vertebral length (VL) were measured in the sagittal view (Figure 1B). Then, we improved the CSDP according to the data measured above (Figure 1C).

### 2.7 Biomechanical stability and safety of cervical subtotal discectomy prosthesis in non-human primates

#### 2.7.1 Construction of non-human primate cervical subtotal discectomy prosthesis implantation model

Based on the cervical anatomical data of the micro-CT measurements above, the dimensions of the CSDP were improved to conform to the vertebral and intervertebral sizes of cynomolgus monkeys for the subsequent implantation. Prior to surgery, anesthesia was induced by intramuscular injection of ketamine (4–6 mg/kg). After tracheal intubation, the ventilator was connected to assist breathing and maintain anesthesia with isoflurane. The monkeys were then placed in the supine position with the surgical area shaved and sterilized, and then the surgical drape was laid. The entire operation was performed in strict accordance with the principles of surgical asepsis. Through the anterior approach, a 3-cm right-sided longitudinal incision was made, and the muscles were bluntly separated to expose the vertebra and C5–C6 intervertebral disc. Before CSDP implantation, the surgical segment was examined using intraoperative radiography. To establish a non-human primate model, a groove through the C6 vertebral body and close to the upper end plate was made by a high-speed burr and curettage, followed by screwing in the CVF structure. Then, the annulus fibrosus and nucleus pulposus were removed with the total upper endplate and partial lower endplate preserved. Finally, after being embedded with the link structure, the CDP structure was implanted at C5–C6 and tightly fixed to the CVF structure by the locking screw to complete the CSDP implantation (Figure 1D). Following disinfection and irrigation with iodophor and normal saline, the incision was layer-sutured and closed.

For the first 3 days post-operation, cefotaxime sodium (50 mg/kg, IM, twice a day) was administered to prevent infection, and rotundine (3 mg/kg, IM, twice a day) was administered to relieve acute pain in surgical animals.

#### 2.7.2 Evaluation of physiological indices in non-human primate model

To further estimate whether CSDP was toxic to non-human primates after implantation, peripheral whole blood was collected for physiological index tests at 1, 3, 7, and 12 months post-surgery. All cynomolgus monkeys were fasted and water-deprived for 12 h before blood collection. After anesthesia was completed with ketamine and the skin was disinfected with iodophor, peripheral whole blood (4 ml) was phlebotomized from the superficial vein of the right calf and preserved in an anticoagulant collection tube. Then, a KX-21N automatic blood analyzer was used for analysis. The blood physiological indices included RBCs, hematocrit (HCT), WBCs, hemoglobin (HGB), mean corpuscular hemoglobin concentration (MCHC), PLTs, lymphocytes (lymphs), neutrophils (OTHRs), and eosinophils (Eos). The blood levels of ALT and AST were used as liver function markers, while BUN was used as a kidney function marker.

#### 2.7.3 Radiology observation

To better understand the osseointegration, displacement, or sinking of CSDP, and surgery-related heterotopic ossification, thin-section (l mm) CT scans (Siemens Munich, Germany) were performed 1 month before and 3, 12, and 18 months after surgery. Meanwhile, a 2.0 MRI scanner (Siemens, Munich, Germany) was used to observe spinal cord or nerve compression, vertebral inflammation, and adjacent-disc degeneration related to the CSDP prosthesis.

#### 2.7.4 Kinematics analysis

Functional impairment of the cervical spinal cord, a high spinal cord segment, could affect the motion of the limbs, which is reflected in gait. Kinematic analysis, a widely used method for recording joint motions in a variety of neuropathological conditions and which enables multi-planar and multi-dimensional motion assessment, is considered one of the most valuable methods for motor function evaluation in quadruped animal models (Diogo et al., 2021). To better understand the potential effect of CDSP implantation on cervical spine function in cynomolgus monkeys, quadruped locomotion was detected 1 month before and 3, 6, 12, and 18 months after surgery.

Kinematic analysis was performed using a gait analyzer (Kinematracer, Kissei Comtec Co., Ltd., Japan) consisting of four stereo cameras with an image resolution of 640 × 480, a motion device, and a terminal machine for data acquisition and analysis. The motion device was a speed-adjustable treadmill that exceeded the maximum reach of the cynomolgus monkey. The gait parameters included the motion trajectory, ROM, stride length, and stance phase ratio of the major joints of the limbs, including the elbows, hips, knees, and ankles. Before the kinematic analysis, the animals were trained to walk on all fours three times a week until they fully cooperated pre- and post-operation. The joint markers, approximately 30 mm in diameter, were sprayed with different colors of non-reflective spray paint for the automatic identification of the joint coordinates in the subsequent analysis. After marking the position dots at the iliac spine and at joints of the hip, knee, ankle, toe, shoulder, elbow, and wrist in different colors, the animals were allowed to walk at a speed of 3 km/h. In total, 10 consecutive cycles were collected for each walk by four cameras, captured at a frame rate of 60 fps. Thereafter, the gait data were imported into 3D-Calculator, and the 3D stick plot models were calculated and reconstructed based on the real-time 3D coordinates of each marked point. These models were subsequently processed using the motion analysis software KineAnalyzer. In KineAnalyzer, a time period containing 10 consecutive motion cycles was selected, the limb contact and departure times were marked, and then the vector directions of the joint lines were determined. All joint angles were defined as spatial angles. Setting the knee and elbow as observation points, the motion ranges were obtained by calculating the angle changes during the gait motion process. Finally, the joint trajectory, stride length, and stance phase ratio of the limbs were obtained based on the average calculation for each marked point.

### 2.8 Statistical analysis

GraphPad Prism 5.0 and SPSS 20.0 were used to process the images and perform the statistical analysis. The results are expressed as the mean ± standard deviation ( $\bar{x}\pm s$ ). The comparison between the two groups, Ti6Al4V-AO and Ti6Al4V, was analyzed using an independent-samples Student’s *t*-test. Differences between multiple groups were analyzed using a one-way analysis of variance. Statistical differences are marked as follows: *p < 0.05, **p < 0.01, ***p < 0.001, n.s., not statistically significant.

## 3 Results

### 3.1 Measurement of cervical spine in non-human primates

Anatomical data on the cervical spines of non-human primates is the basis for improving the CSDP to match an individual’s size. To this end, we first performed 3D CT measurements of the cervical spine before designing CSDP implantation in cynomolgus monkeys. In general, increasing trends were observed in both the intervertebral disc space from C2/C3 to C6/C7 and the vertebra size from C2 to C7 (Table 1). Meanwhile, C6 and C7 demonstrated larger and similar sizes of the vertebral body and the upper intervertebral space. Considering the cervical mobility and the difficulties of operation and radiological observation, the C6 and upper intervertebral discs were selected as the surgical segment; this happens to be the segment with the most cervical intervertebral disc lesions in humans.

**TABLE 1:** Cynomolgus monkey cervical spine anatomical measurements.

Segment	Intervertebral space (mm)				Ordinal	Vertebra (mm)	
	IAEW	IPEW	ILEW	IH		VH	VL
C2/C3	6.21 ± 1.04	3.58 ± 0.82	5.64 ± 1.09	2.26 ± 0.23	C3	6.21 ± 0.68	7.10 ± 0.43
C3/C4	7.12 ± 0.41	4.29 ± 0.79	5.80 ± 0.81	2.20 ± 0.08	C4	5.72 ± 0.41	6.80 ± 0.21
C4/C5	6.81 ± 0.81	4.15 ± 0.73	6.21 ± 0.60	2.29 ± 0.10	C5	5.94 ± 0.29	7.15 ± 0.62
C5/C6	7.11 ± 0.94	4.49 ± 0.40	6.26 ± 0.64	2.33 ± 0.05	C6	6.38 ± 0.50	7.56 ± 0.43
C6/C7	7.72 ± 0.86	4.76 ± 0.43	6.34 ± 0.75	2.23 ± 0.06	C7	6.39 ± 0.29	8.16 ± 0.88

IAEW, intervertebral anterior edge width; IPEW, intervertebral posterior edge width; ILEW, intervertebral lateral edge width; IH, intervertebral height; VH, vertebral height; VL, vertebral length.

The dimension of the CSDP was successfully improved, with the CVF structure at a size of 4.5 mm in diameter and 8.5 mm in length, while the CDP structure was adapted with 7 mm anterior edge width, 4 mm posterior edge width, and 6 mm lateral length, which was suitable for most segments of the cervical vertebra and intervertebral space of cynomolgus monkeys (Figure 1C).

### 3.2 Cytocompatibility evaluation of cervical subtotal discectomy prosthesis *in vitro*

The cell morphology, proliferation, and cytotoxicity of MC3T3-El cells co-cultured with Ti6Al4V-AO were evaluated for the cytocompatibility detection of the CSDP.

#### 3.2.1 Observation of cell morphology

**FIGURE 2:**
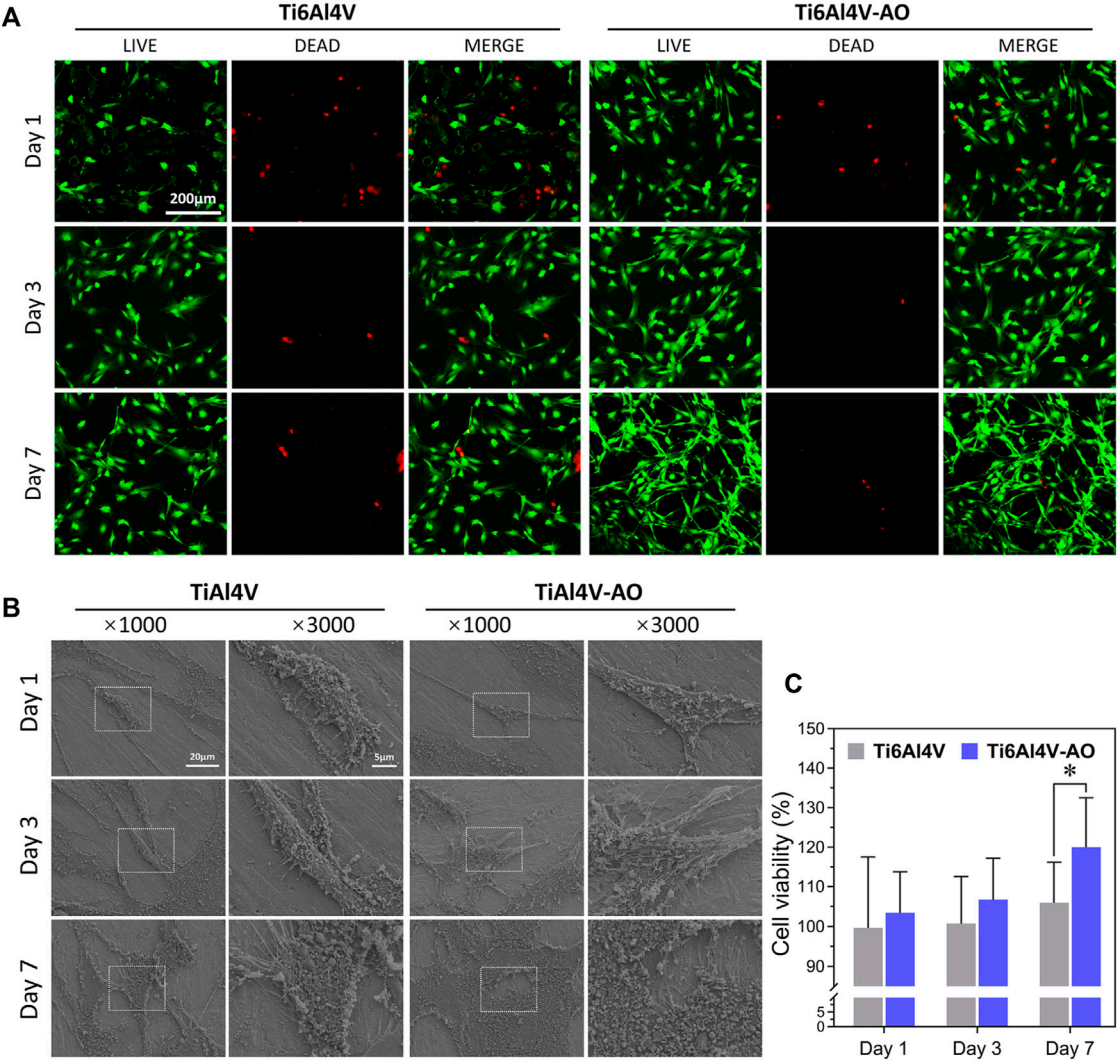
Biocompatibility detection of CSDP *in vitro*. **(A)** LIVE/DEAD cell viability observation. The cell viability of MC3T3-E1 cells co-cultured with Ti6Al4V-AO and Ti6Al4V for 1, 3, and 7 days. **(B)** Cell adhesion morphology scanning electron microscopy observation. The adhesive cell morphology of MC3T3-E1 cells co-cultured with Ti6Al4V-AO and Ti6Al4V for 1, 3, and 7 days. **(C)** CCK-8 cell proliferation rate detection. The proliferation rate of MC3T3-E1 cells co-cultured with Ti6Al4V-AO and Ti6Al4V for 1, 3, and 7 days. (*n* = 4). *p < 0.05.

At day 1, no significant differences in cell density were observed on the surfaces of the Ti6Al4V-AO and Ti6Al4V metal discs, but the cell morphology was more extended in the Ti6Al4V-AO group. When co-cultured for 3 days, the cells adhered with a large number of pseudopods to the surface of all samples, showing good growth and increased cell density. The cells were observed to overlap and spread on the surfaces of all samples, with significantly increased cell density at the 7th day. However, the MC3T3-El cells tiled more extensively on the surface of the Ti6Al4V-AO metal discs and had more pseudopods than those in the Ti6Al4V group, indicating the superior cell adhesion capability of the CSDP material (Figure 2B).

#### 3.2.2 Cell proliferation assay

On days 1, 3, and 7 of co-cultivation, the cell proliferation rates of the Ti6Al4V-AO group were more than l00%, and they were much higher than those of the Ti6Al4V group (Figure 2C). The differences were statistically significant. This shows that the CSDP has a positive effect on cell proliferation.

#### 3.2.3 Cytotoxicity detection

In addition to detecting the cell adhesion and proliferation capabilities of the CSDP, we performed LIVE/DEAD staining as a marker for cytotoxicity detection. When co-cultured with Ti6Al4V-AO for 1, 3, and 7 days, the MC3T3-El cells presented an extended morphology and increased quickly. Compared with the control group, there were many more viable cells in the Ti6Al4V-AO group, with few dead cells observed (Figure 2A), which further demonstrates that the CSDP has minimal cytotoxicity.

### 3.3 Evaluation of biocompatibility and osseointegration of cervical subtotal discectomy prosthesis *in vivo*

Although the safety of the CSDP material was verified at the cellular level, we additionally developed a femoral intramedullary nail-implantation mouse model to verify its biocompatibility and osseointegration capacity *in vivo* before the CSDP was formally implanted into the primate cervical spine.

#### 3.3.1 Biocompatibility evaluation of cervical subtotal discectomy prosthesis in mouse model

**FIGURE 3:**
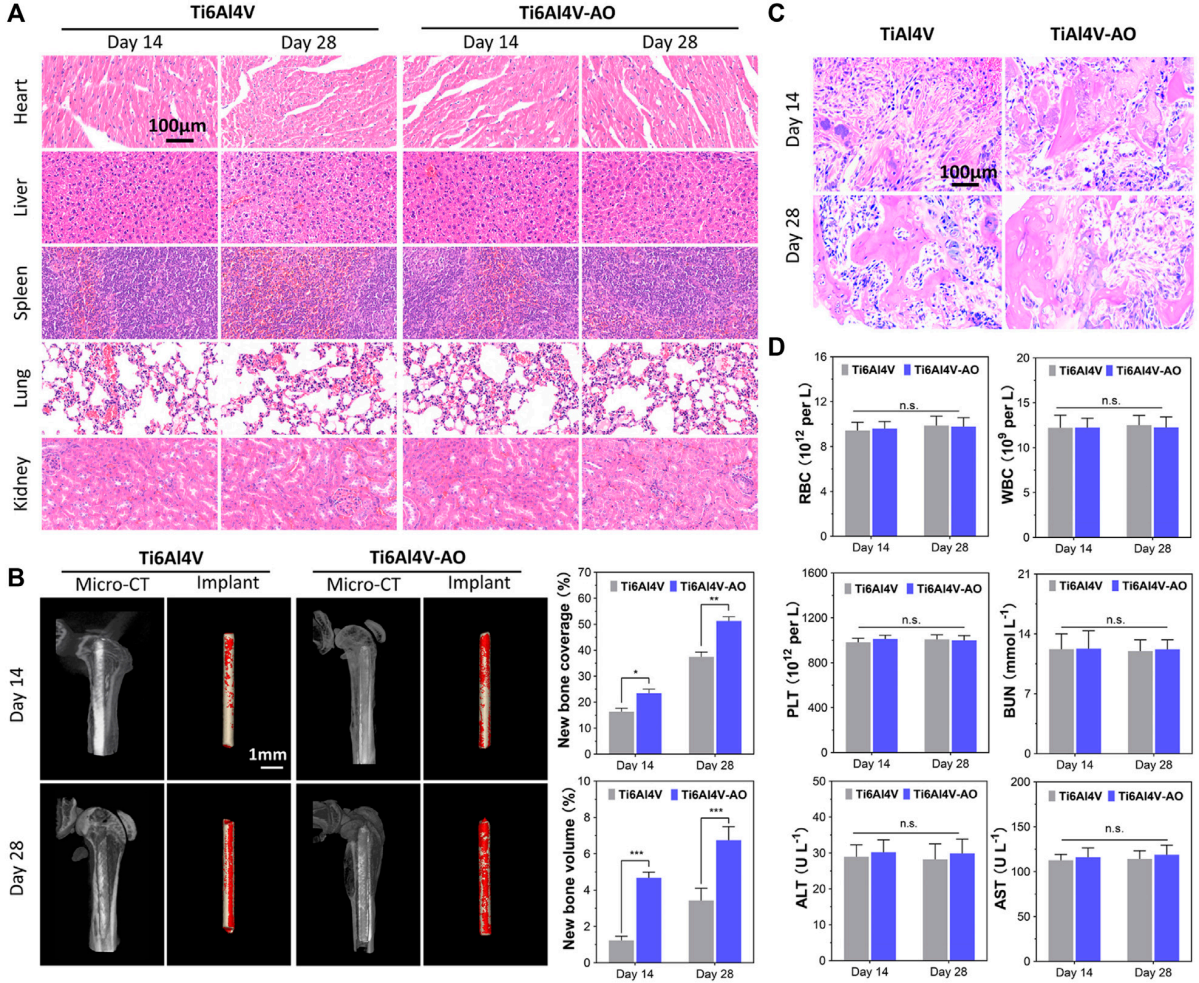
Evaluation of biocompatibility and osteogenesis of CSDP in mouse model. **(A)** Hematoxylin and eosin (HE) staining of mouse organs at 14 and 28 days after surgery. **(B)** Osteogenesis morphological analysis of micro-CT in mouse model. **(C)** HE staining of bone tissue sections around implants. **(D)** Mouse blood biochemical test. The complete blood data, including white blood cells (WBCs), red blood cells (RBCs), and platelets (PLTs). Blood levels of alanine aminotransferase (ALT) and aspartate aminotransferase (AST) as liver function markers. Blood urea nitrogen (BUN) as kidney function marker. *p < 0.05, **p < 0.01, ***p < 0.001, n.s., no statistical difference (*n* = 12).

At 14 and 28 days post-operation, the mice showed satisfactory motor function, and their blood physiological indices were all within the normal range, with no significant differences between the groups (Figure 3D). The detection of normal ranges of WBCs, RBCs, and PLTs indicated that no obvious inflammatory reactions occurred, while normal ALT, AST, and BUN levels revealed that the metal material of the CSDP caused no obvious hepatotoxicity or nephrotoxicity to the mice. In addition, no pathological changes were observed in the pathological sections of the heart, liver, spleen, lungs, or kidneys (Figure 3A).

#### 3.3.2 Osseointegration observation

The overall observation of 3D reconstruction images of the femurs suggested that no infection, osteolysis, or osteonecrosis occurred around the implants in either group, at 14 or 28 days post-operation. As early as 14 days post-operation, new cancellous bone (red) was formed on the surface of the implant, and bone connection between the metal pin implants and the femoral cortical bone was observed. However, both the coverage and the volume of new bone on the pins of the Ti6Al4V-AO group were significantly more extensive than those in the control group (*p* < 0.05, *n* = 12) (Figure 3B). Growing coverage of new cancellous bone in both groups was observed 28 days after surgery, but the increase was more obvious on the surface of Ti6Al4V-AO. Additionally, new bone trabeculae were observed around all implants in the pathological sections of the femur, and the structure of the new bone trabeculae was clearly displayed. However, the number and density of new bone trabeculae around the Ti6Al4V-AO metal pins were better than those in the control group, at both 14 and 28 days post-operation (Figure 3C).

These positive results suggest that the metal composition of the CSDP has a satisfactory capacity for osseointegration and biocompatibility.

### 3.4 Safety assessment of cervical subtotal discectomy prosthesis implanted in non-human primate model

Previously, the biocompatibility and bone integration of the constituent material of the CSDP were preliminarily proved by assessments at the cellular level and in a mouse model. We constructed a non-human primate model of CSDP implantation and verified the safety of the final product in cynomolgus monkeys. No significant dimensional mismatch was observed during the surgical implantation of the CSDP.

**FIGURE 4:**
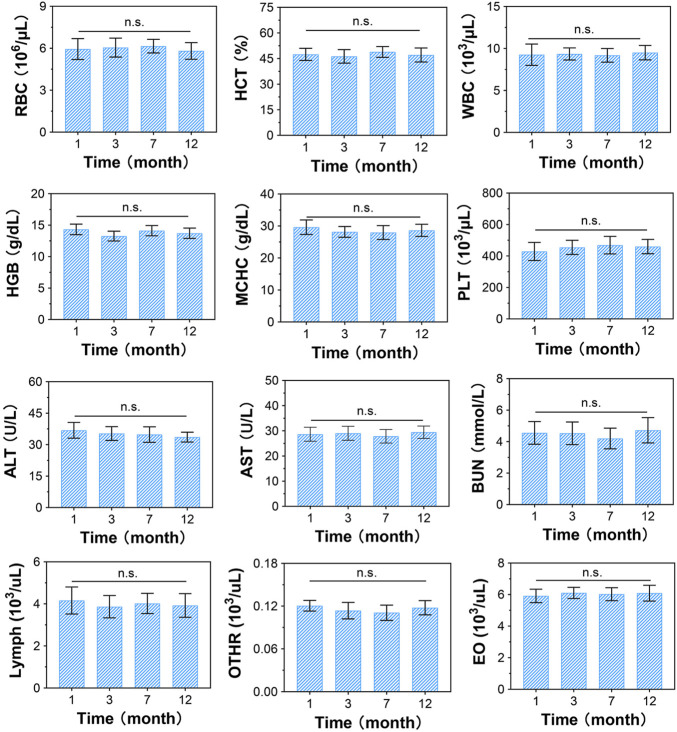
Blood biochemical evaluation of CSDP implanted in non-human primate model. The blood routine includes red blood cells (RBCs), hematocrit (HCT), white blood cells (WBCs), hemoglobin (HGB), mean corpuscular hemoglobin concentration (MCHC), platelets (PLTs), lymphocytes (lymphs), neutrophils (OTHRs), and eosinophils (EOs). Blood levels of alanine aminotransferase (ALT) and aspartate aminotransferase (AST) used as liver function markers. Blood urea nitrogen (BUN) used as kidney function marker. n.s., no statistical difference (*n* = 8).

At 1, 3, 7, and 12 months post-surgery, all monkeys had normal vital signs and motor function, and their physiological indices were all within the normal range (Figure 4). The normal detection of RBCs, WBCs, PLTs, HGB, HCT, MCHC, lymphs, OTHRs, and EOs indicated that there were no obvious side effects on the blood system, while normal ALT, AST, and BUN levels suggested that the CSDP caused no hepatotoxicity or nephrotoxicity in the surgical monkeys. These results revealed that the CSDP had no significant side effects on the physiological function of the non-human primate model after implantation.

### 3.5 Radiological observation of the osseointegration and biomechanical stability of cervical subtotal discectomy prosthesis

Radiological observation is a direct and crucial evaluation method for animal models of prosthesis implantation. Thus, the postoperative osseointegration and biomechanical stability of the CSDP were evaluated by X-ray, CT, and MRI observations of the primate cervical spine at 3, 12, and 18 months post-surgery.

#### 3.5.1 X-ray results

**FIGURE 5:**
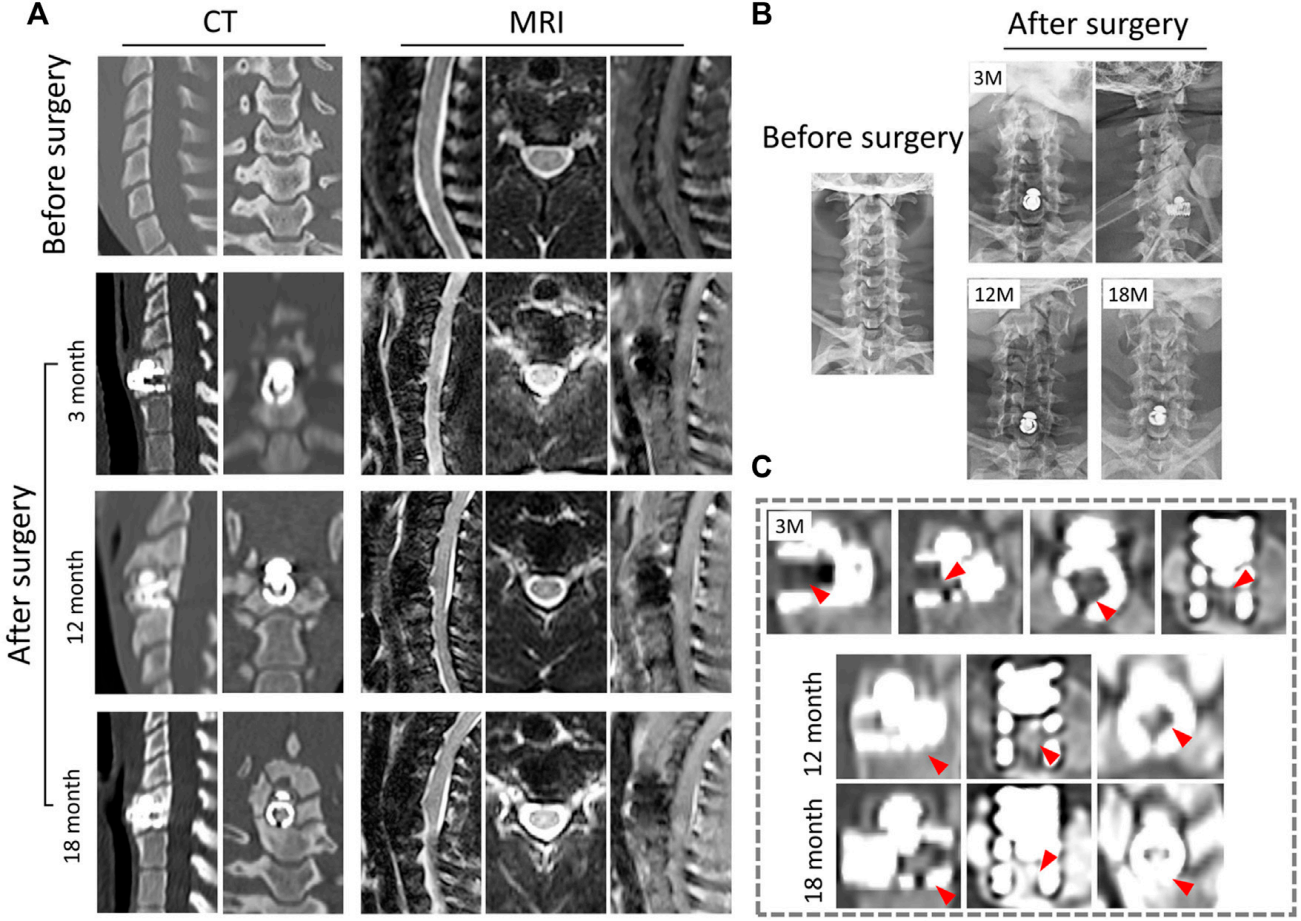
Radiological observation of CSDP in non-human primates. **(A)** CT and MRI 1 month before and 3, 12, and 18 months after CSDP implantation. **(B)** X-ray imaging 1 month before and 3, 12, and 18 months after CSDP implantation. **(C)** The trabecular bone gradually grew into the cervical vertebra fixation (CVF) structure through the tunnel at 3 months post-operation. Increased quantity and density of trabecular bone grew into the CVF structure, and the CSDP obtained intravertebral fusion at 1 year, then strengthened at 18 months post-surgery.

The X-ray radiographs revealed that no significant changes occurred in the physiological curvature of the cervical spine after CSDP implantation. Furthermore, no displacement of the implants was observed in the 18-month follow-up (Figure 5B).

#### 3.5.2 CT imaging

In general, there was no sinking or dislocation of the CSDP, nor any infection, osteolysis, or osteonecrosis in any of the implant locations within 18 months, according to follow-up CT scans of the cervical spine (Figure 5A). Meanwhile, a crucial sign of bony fusion was observed, in that the new trabecular bone gradually grew into the CVF structure by 3 months. At 1 year post-operation, the internal cavity of the CVF structure was filled with new bone, and intravertebral fusion and fixation of the CSDP were achieved. In addition, no severe operation-related heterotopic ossification was found in the operative segment. At the 18-month post-operation follow-up, in addition to the presence of a larger bony enclosure around the implants, the density of the cancellous bone in the CVF structure was further increased, and the CSDP had obtained a more stable intravertebral fusion (Figure 5C).

#### 3.5.3 MRI observation

In the 18-month follow-up, no compression of the spinal cord or nerve root was exhibited, nor were adjacent-segment degeneration, inflammation, or spinal cord edema observed in any of the MRI observations (Figure 5A). Excision of the cervical structure and prosthesis implantation can affect the mechanics of the cervical spine to a certain extent, which may lead to biomechanical instability. However, in this study, no obvious changes in the cervical force line were observed. Signs of biomechanical instability, such as prosthesis dislocation and hypermobility, were not assessed in the cervical hyperextension MRI examination at 18 months post-operation.

### 3.6 Neurological effect evaluation of cervical subtotal discectomy prosthesis after implantation

After verifying the safety and stability of the CSDP in a non-human primate model, we conducted a postoperative kinematic analysis of cynomolgus monkeys to evaluate the effect of the CSDP on cervical spinal nerve function.

**FIGURE 6:**
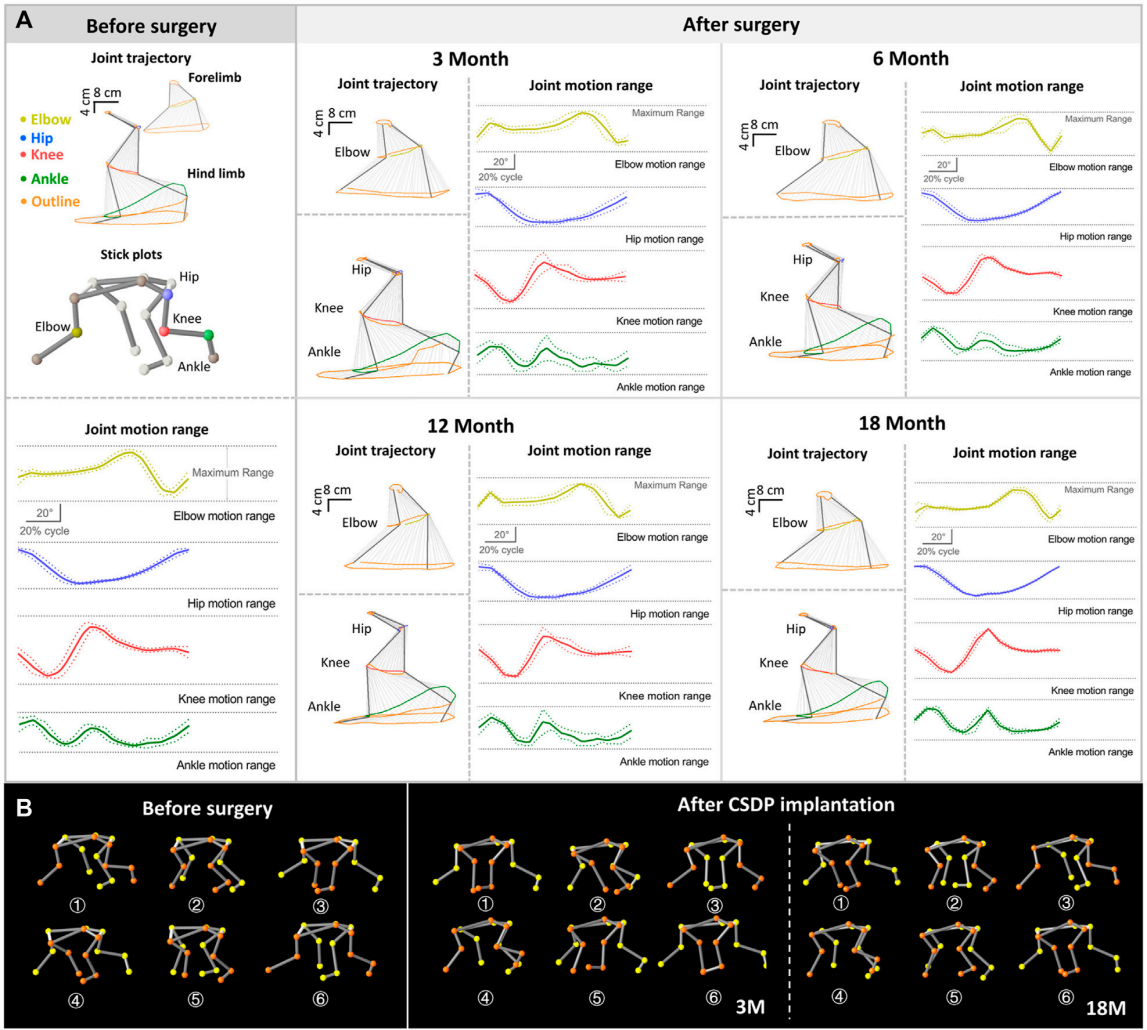
Kinematic analysis of limbs and joint trajectory situation in non-human primates. **(A)** The joint trajectory and joint motion range analysis at 1 month before surgery and 3, 12, and 18 months after surgery. **(B)** The kinematic analysis 3D-stick plot models pre and post-operation, describing the basic situation of the joint trajectory.

**FIGURE 7:**
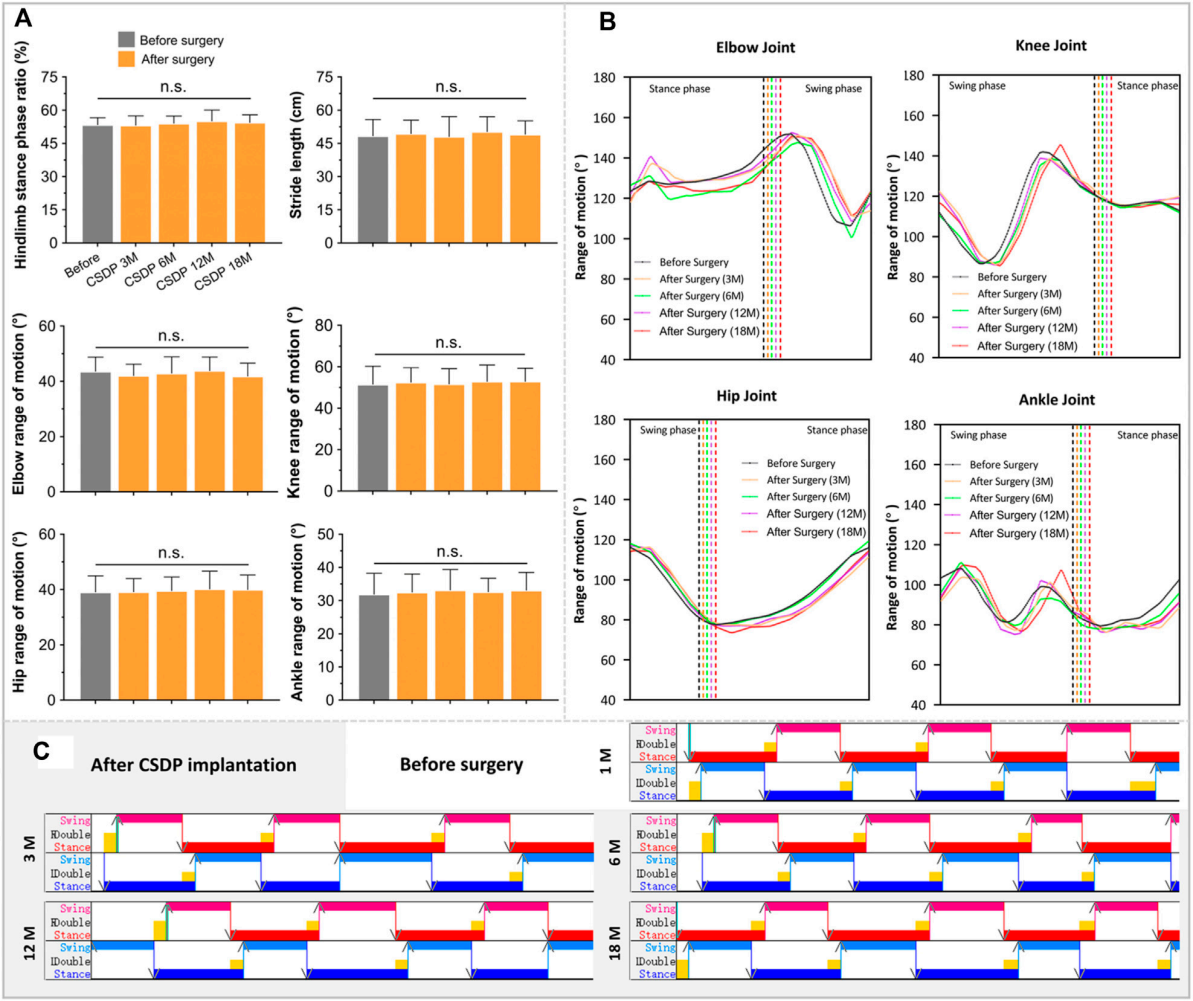
The foundation date of kinematic analysis in non-human primates. **(A)** Quantitative detections of stance phase ratio, stride length, and limb range of motion of elbow, hip, knee, and ankle. **(B)** The basic statistics of changes in joint trajectory values at 1 month before surgery and 3, 6, 12, and 18 months after surgery. **(C)** Visualized strip-based data of forelimb and hindlimb stance and swing phases. n.s., no statistical difference. (*n* = 8).

The walking cycle consisted of a stance phase and a swing phase, which are performed differently by the forelimbs and hindlimbs of cynomolgus monkeys, but the walking posture and ROM did not change after surgery in the stick plots (Figures 6B, 7B). Initially, the passive activities of the limbs were driven by rotation of the motion device, resulting in irregular motion trajectories. However, the monkeys began to walk autonomously after a short period of adaptation (Figure 7C). In the quantitative detections of the joint trajectory, the gait consistency was excellent (Figure 6A), and no statistical differences were found in limb ROM pre- and postoperatively at 3, 6, 12, or 18 months (Figure 7A). Moreover, compared to preoperative values, no statistical changes in stride length or stance phase ratio were observed in the 18-month follow-up (Figure 7A). In addition, no obvious pathological changes were observed in the motion trajectory of the forelimbs and hindlimbs post-surgery (Figure 6B).

The kinematic analysis indicated that peripheral nerve function was in good condition after CSDP implantation and that the CSDP had no adverse effects on cervical spinal cord function.

## 4 Discussion

Preserving as much cervical mobility as possible and reducing the incidence of adjacent segment disease (ASD) are crucial considerations for neurosurgeons in surgical planning. In contrast to anterior cervical discectomy and fusion (ACDF), artificial cervical disc replacement (ACDR) can maintain a certain mobility of the cervical spine after surgery and has gained widespread attention and clinical applications (Coban et al., 2021). The construction of ball-and-socket joints, whether metal-to-metal or metal-to-plastic, is regarded as the key to ensuring mobility for artificial cervical discs and is currently the mainstream design (Choi et al., 2017). However, the excessive ROM and rigid connection between the metal articular surfaces brought about by the design concept could increase the pressure on the facet joints (Lee et al., 2011), with long-term overload leading to surgical segment degeneration and ASD, which increases the risks of prosthesis subsidence and dislocation (Coban et al., 2021). Heterotopic ossification associated with prosthesis implantation is also considered a challenging problem that cannot be ignored (Dey et al., 2017; Wang et al., 2018).

In this study, considering these complications, we designed a new type of prosthesis for cervical subtotal discectomy (CSDP) based on a concept similar to hemi-arthroplasty. Before implantation, we first verified that the CSDP possessed satisfactory biocompatibility at the cellular level; then, the mouse model construction of the femoral intramedullary nail suggested that the CSDP had favorable osseointegration ability. After improving the size according to the cervical spine anatomical measurements of cynomolgus monkeys, a non-human primate model of CSDP implantation was constructed. Physiological index detection further proved that the prosthesis possessed good histocompatibility and safety. Upon pre- and post-operation examination, excellent biomechanical stability of the CSDP after implantation was indicated in an 18-month follow-up radiology observation. Finally, kinematic analysis demonstrated that the CSDP had no adverse effects on cervical nerve function.

The key to retaining the motion function of the CSDP is the construction of semi-restricted ellipsoid-in-socket articulation. This newly designed metal-to-polyethylene joint is designed to diminish the collisions of the previously used metal-to-metal articulus and to reduce attrited metal particles which might potentially impact the physiological microenvironment (Stojanovic et al., 2019). In addition to ensuring a certain physiological ROM of the cervical spine, this articulation limits hypermobility, which further reduces the risk of prosthesis prolapse.

The CSDP was designed to retain the lower endplate during implantation. The long-term stability of the prosthesis mainly depends on the bony fusion of the CVF structure and vertebral body. Therefore, it is crucial to evaluate the overall stability of the CSDP in implantation models of large animals. To date, large animals, such as goats, sheep, and primates, have been widely used as models for the construction of artificial disc prostheses (Kotani et al., 2002; Zhang et al., 2021). Non-human primates are considered the most desirable models for the unique characteristics of humanoid genetics (Rao et al., 2018), anatomy, and, with their habit of climbing and upright activities, spinal biomechanics. However, given the size differences between cynomolgus monkeys and humans, adaptive improvement of the CSDP should be made before implantation.

Detailed and accurate cervical anatomical data for primates are the basis for the design, development, and personalized customization of artificial cervical intervertebral discs. In addition to traditional specimen measurement of the cervical spine, the CT scan is another major anatomical measurement technique (Phal et al., 2008). It avoids the dehydration of muscles and ligaments and poor display of bone structure caused by long-term fixation and can clearly reveal the tiny bone structure and facet joint (Michael et al., 2013). In this study, 3D micro-CT was performed to survey the steric parameters of the cervical spine in different reconstructed planes, based on the reverse modeling of the spiral scanning date, which is considered more free and accurate (Andresen et al., 2015) than conventional CT. Significantly, its detailed and precise measurement directly helped us to successfully implant the CSDP in the cervical spine of non-human primate models.

In our previous research on CSDP finite element analysis (Wo et al., 2021), we found that force can be well-transmitted without excessive stress concentration from the prosthesis to the lower bone cortex through the connection structure and CVF structure. Meanwhile, the facet joint can maintain a stable load in motion and reduce the compensatory movement demand of the adjacent segment. In addition, simulation wear evaluation proved that the CSDP was stable and durable. However, the firm combination of the titanium CVF structure and vertebra is the foundation and guarantee of the overall mechanical stability of the CSDP.

The nano-oxide layer of titanium alloy modified by anodic oxidation can effectively improve biocompatibility (Ali et al., 2011; Wang et al., 2014), and this material in the CVF structure achieved satisfactory performance both at the cellular level and with *in vivo* validation in this study. In addition, after implantation into the mouse femur, the surface of the modified titanium alloy was quickly covered by new cancellous bone and widely connected to the surrounding cortical bone, indicating that the CVF structure has excellent osseointegration ability.

Although the CSDP showed good mechanical stability in finite element analysis, and its biocompatibility and osteogenic ability were verified at the cellular level and in a mouse model, the overall safety and stability of the CSDP in a non-human primate model still needs to be further studied before its clinical application.

After implantation, physiological examination of the blood indicated that the CSDP produced no tissue toxicity. Exogenous artificial intervertebral discs may suffer from sinking, displacement, and compression of the spinal nerves due to poor design, stiffness disparity at the bone interface, premature weight-bearing, and insufficient support of muscles and ligaments. Fortunately, these can be detected promptly by early radiological observation. Under stable mechanical conditions, the long-term stability of the artificial disc depends on the osseous fusion of the metal-bone interface.

In general, the bone around the implant stress line could be absorbed at 3 months post-operation owing to prosthesis fretting caused by unstable mechanics, which would indicate failure of the surgery (Rahimizadeh et al., 2018). Timely immobilization and force adjustment are helpful in preventing this treatment outcome in subsequent bony fusion. Strong osteogenesis in and around the prosthesis is a sign of biological bone stability. In this study, the CVF structure was gradually filled with new bone, and no sinking or displacement of the prostheses was observed during the 18-month follow-up CT scans, indicating that the CSDP achieved bone fusion and fixation. It also proved that the CSDP had the advantage of good mechanical stability after surgery, which provided sufficient conditions for osseointegration and the final ingrowth of cancellous bone in the initial stage of CSDP implantation. In addition, no heterotopic ossification or obvious hyperosteogeny were found post-surgery, which may be due to the good stability of the CSDP, which reduces local fretting and weakens reactive bone hyperplasia.

Implantation of an exogenous artificial cervical disc and the operation itself destroy the original cervical structure and stability to a certain extent, resulting in possible neurological symptoms after surgery (Loidolt et al., 2021). MRI can sensitively detect implantation-related complications such as degeneration, ASD, and spinal cord compression caused by prosthesis displacement and heterotopic ossification. It is critical to evaluate the safety and stability of artificial intervertebral disc implantation in large animals. After 1 year of post-surgical follow-up, no spinal cord compression in the surgical or adjacent segments was observed on MRI, nor was degeneration of the adjacent intervertebral disc or vertebral inflammatory reaction found, which further verified that the CSDP had good mechanical stability and safety after implantation. This was also reflected in the negative results of the four blood biochemical tests at 1, 3, 7, and 12 months post-operation. Long-term implantation of the CSDP did not damage the blood system, immune system, liver, or kidney functions.

Cervical instability symptoms, such as hypermobility and spondylolisthesis, are manifestations of declining cervical spine function, which affects the state of the cervical spinal cord but may appear negative in early radiology observations (Wei et al., 2016; Zhao et al., 2017). As a high spinal cord segment, functional impairment of the cervical spinal cord affects the motor state of the limbs, which can be reflected in the gait at an early stage. Kinematic analysis is a quantitative and highly accurate method for detecting small abnormal gait changes in animals that have undergone surgery (Diogo et al., 2019). However, effective kinematic analysis for CSDP requires good cooperation of monkeys; therefore, cooperative walking training should be performed before surgery. Obtaining objective gait results within 2 months after surgery is difficult because of the unstable operation area of the cervical spine, and forced detection may cause additional damage to monkeys and affect follow-up observations. At 3 months post-surgery, we observed no abnormalities in gait parameters of joint trajectory, knee and ankle joint motion range, stride length, or stance phase ratio, indicating that the CSDP had no significant effect on cervical spine function after implantation. This was exactly when the ingrowth of new bone in the CVF structure was also observed in the CT scan and the bony fusion process of the CSDP began. No gait changes were observed in the subsequent 6- and 12-month long-term follow-ups, which further proved the satisfactory safety of the CSDP after implantation.

In this study, we constructed a successful non-human primate CSDP implantation model for the evaluation of artificial cervical discs and proved that it has excellent osseointegration capacity, safety, and biomechanical stability. However, under certain circumstances, follow-up research is necessary. Unlike humans, non-human primates cannot directly, verbally communicate their discomfort symptoms to researchers, and spinal cord-related symptoms that have not yet caused gait changes are currently undetectable but may be helpful for prosthetic evaluation. Second, only a single-segment operation was performed in this study, and the evaluation of continuous or multi-segment CSDP implantation needs to be performed in a subsequent study. Additionally, the animals that underwent surgery have not been harvested, and post-surgery immunological and histopathological evaluations have not been performed. The evaluation of the long-term implantation effects of the CSDP requires a follow-up study.

## 5 Conclusion

In conclusion, we designed a new CSDP and successfully constructed an excellent non-human primate implantation model for the evaluation of subtotal disc replacement arthroplasty. Furthermore, we demonstrated that the CSDP had outstanding safety, osseointegration capacity, and biomechanical stability in a non-human primate model. This study aimed to explore the effectiveness of an alternative to traditional cervical interbody fusion surgery, providing a novel artificial cervical disc replacement concept that may become a new treatment option for degenerative cervical diseases.

## Data Availability

The original contributions presented in the study are included in the article and supplementary material. Further inquiries can be directed to the corresponding authors.
